# Decreased Ecological Resistance of the Gut Microbiota in Response to Clindamycin Challenge in Mice Colonized with the Fungus Candida albicans

**DOI:** 10.1128/mSphere.00982-20

**Published:** 2021-01-20

**Authors:** Laura Markey, Antonia Pugliese, Theresa Tian, Farrah Roy, Kyongbum Lee, Carol A. Kumamoto

**Affiliations:** a Graduate School of Biomedical Sciences, Tufts University School of Medicine, Boston, Massachusetts, USA; b Department of Chemical and Biological Engineering, Tufts University School of Engineering, Medford, Massachusetts, USA; c Department of Biostatistics, Harvard T.H. Chan School of Public Health, Boston, Massachusetts, USA; d Department of Molecular Biology and Microbiology, Tufts University School of Medicine, Boston, Massachusetts, USA; University of Georgia

**Keywords:** *Candida albicans*, ecological resistance, microbiota

## Abstract

The mammalian gut microbiota is a complex community of microorganisms which typically exhibits remarkable stability. As the gut microbiota has been shown to affect many aspects of host health, the molecular keys to developing and maintaining a “healthy” gut microbiota are highly sought after. Yet, the qualities that define a microbiota as healthy remain elusive. We used the ability to resist change in response to antibiotic disruption, a quality we refer to as ecological resistance, as a metric for the health of the bacterial microbiota. Using a mouse model, we found that colonization with the commensal fungus Candida albicans decreased the ecological resistance of the bacterial microbiota in response to the antibiotic clindamycin such that increased microbiota disruption was observed in C. albicans*-*colonized mice compared to that in uncolonized mice. C. albicans colonization resulted in decreased alpha diversity and small changes in abundance of bacterial genera prior to clindamycin challenge. Strikingly, co-occurrence network analysis demonstrated that C. albicans colonization resulted in sweeping changes to the co-occurrence network structure, including decreased modularity and centrality and increased density. Thus, C. albicans colonization resulted in changes to the bacterial microbiota community and reduced its ecological resistance.

**IMPORTANCE** Candida albicans is the most common fungal member of the human gut microbiota, yet its ability to interact with and affect the bacterial gut microbiota is largely uncharacterized. Previous reports showed limited changes in microbiota composition as defined by bacterial species abundance as a consequence of C. albicans colonization. We also observed only a few bacterial genera that were significantly altered in abundance in C. albicans-colonized mice; however, C. albicans colonization significantly changed the structure of the bacterial microbiota co-occurrence network. Additionally, C. albicans colonization changed the response of the bacterial microbiota ecosystem to a clinically relevant perturbation, challenge with the antibiotic clindamycin.

## Abstract (precis)

Candida albicans is the most common fungal member of the human gut microbiota, yet its ability to interact with and affect the bacterial gut microbiota is largely uncharacterized. Previous reports showed limited changes in microbiota composition as defined by bacterial species abundance as a consequence of C. albicans colonization.

## INTRODUCTION

The human bacterial gut microbiota is a complex ecosystem consisting of hundreds of different bacterial species which interact with each other and with the host to reach a stable long-term equilibrium. Although there is significant variability between individual human hosts, within a host, the composition and diversity of the gut microbiota are remarkably stable over time (1). This high degree of interhost gut microbiota diversity and variability is one reason it has been difficult to define a standard consortium of bacteria as a “healthy” microbiota. Certain characteristics of the gut microbiota such as higher or lower overall diversity (2–4) or the *Firmicutes*/*Bacteroidetes* ratio (5) have been associated with healthy or disease states, yet the molecular mechanisms by which the bacterial microbiota affects host physiology remain largely uncharacterized. Using monocolonization of germfree mice, researchers have defined how commensals (*Lactobacillus* sp. [6, 7] and *Bifidobacterium* sp. [8, 9]) can affect the host and vice versa. However, the key properties of a gut microbiota associated with host health remain undefined.

Candida albicans is the most prevalent human commensal fungus, a common member of the human gut microbiota (10), and the most common fungal opportunistic pathogen of humans (11). C. albicans burden in stool was associated with worse fecal microbiota transplantation (FMT) outcomes (12), and C. albicans was significantly more likely to be isolated from Crohn’s disease patients than from healthy matched controls (13), suggesting an association between the presence of C. albicans in the gut microbiota and gastrointestinal (GI) disease states. In mouse models of GI disease, C. albicans colonization has been associated with both protective (14–16) and detrimental (17–19) outcomes. In this work, we measured the effect of colonization with the fungus Candida albicans on the mouse bacterial fecal microbiota. Consistent with previous observations (14, 20), C. albicans colonization did not substantially alter the composition of the fecal bacterial microbiota, although some changes in genus abundance and diversity were detected.

An ecological network approach has shown that long-term stability and diversity of the human microbiota rest upon interactive networks of cooperation and competition (21), often centered around “keystone” hub species that are highly integrated and central to the network as a whole (22). The interspecies relationships represented by interaction networks of the microbiota are thought to contribute to ecological resistance (23), the ability to maintain an ecosystem in the face of changing external conditions (24). Disruption of these relationships or removal of a keystone species can reduce ecological resistance and lead to destabilization of the network (25), with potential consequences for the host.

In this work, we used network analysis to study the effect of C. albicans colonization on the co-occurrence network of the bacterial microbiota. C. albicans colonization had a significant impact on the co-occurrence network topology. We then analyzed the response of the bacterial community to perturbation with the antibiotic clindamycin to determine whether the differences between bacterial communities in uncolonized versus C. albicans-colonized mice would affect the ability of the community to resist perturbation. Despite the similarities in the composition of the microbiota, the communities responded differently to perturbation with the antibiotic. C. albicans-containing communities were more extensively altered by treatment and thus exhibited lower ecological resistance to this perturbation.

## RESULTS AND DISCUSSION

### C. albicans colonization had modest effects on the diversity and composition of the bacterial microbiota in a cefoperazone pretreatment model.

**FIG 1:**
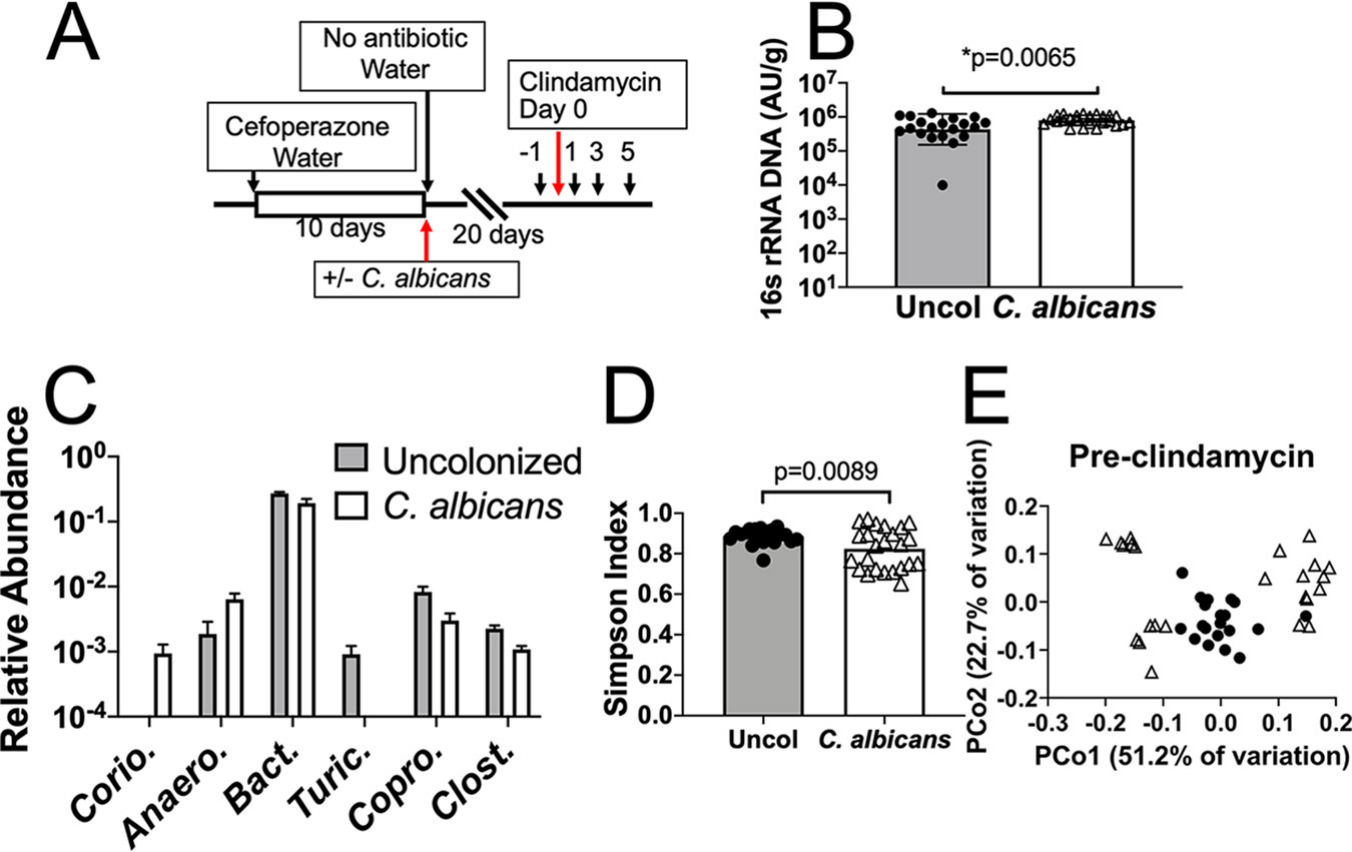
Effects of C. albicans colonization on the composition of the bacterial microbiota. Mice were pretreated with cefoperazone in drinking water for 10 days and then either colonized with C. albicans (5 × 10^7^ CFU by oral inoculation) or not. Mice were then switched back to sterile water, and the microbiota were allowed to recover for 3 weeks. (A) Experimental design timeline. Fecal pellets were collected (black arrows with numbers indicating day of collection) for bacterial microbiota analysis by 16S rRNA sequencing. Mice were from two separate experimental trials; total uncolonized, *N* = 20, and C. albicans colonized, *N* = 24. (B) qPCR of universal 16S rRNA sequence was used to measure the total bacterial DNA content (in arbitrary units) per gram of feces for each sample used for sequencing analysis. (C) Relative abundance of genera in the pre-clindamycin (day −1) microbiota of uncolonized mice and C. albicans-colonized mice. Genera shown were identified as differential and significantly associated with C. albicans colonization status by LEfSe analysis. Bars indicate the averages and error bars the standard errors of the means (SEMs). Corio, unclassified *Coriobacteriaceae*; Anaero, *Anaeroplasma*; Bact, *Bacteroides*; Turic, *Turicibacter*; Copro, *Coprococcus*; Clost, *Clostridiaceae Clostridium*. (D) Alpha diversity of the pre-clindamycin microbiota of uncolonized mice and mice colonized with C. albicans as measured by the Simpson index. Symbols indicate the diversity indices of individual mouse microbiota and bars indicate the averages. (E) Principal-coordinate analysis of the weighted UniFrac distance matrix of the pre-clindamycin bacterial microbiota of uncolonized mice (●) and mice colonized with C. albicans (△).

C. albicans is the most common fungal member of the human gut microbiota, and the extent of its participation in gut microbiota dynamics is poorly understood. In this study, pretreatment with the beta-lactam antibiotic cefoperazone was used to enable stable C. albicans colonization (Fig. 1A), because C. albicans is not a native colonizer of mice. Mice were treated with cefoperazone in drinking water for 10 days and then either colonized with C. albicans (5 × 10^7^ CFU) or not colonized (Fig. 1A). They were then allowed to recover from cefoperazone treatment for 3 weeks. C. albicans colonization levels were monitored by plating fecal pellets for fungal CFU (see Fig. S1B in the supplemental material). No fungal colonies were recovered from fecal pellets from uncolonized mice, and C. albicans was recovered from all colonized mice.

10.1128/mSphere.00982-20.2 FIG S1 Quantitation of bacteria and C. albicans in fecal pellets. (A) qPCR of universal 16S rRNA sequence was used to measure the total bacterial DNA content per sample used for sequencing analysis. Untreated samples were collected one day prior to clindamycin challenge. Total bacterial DNA was also measured in fecal pellets collected one day after clindamycin challenge, from mice challenged with a low (1.11 mg/kg body weight) intermediate (3.33 mg/kg), or high (10 mg/kg) dose of clindamycin. This value in arbitrary units was normalized to the weight of the starting material. (B) Fecal pellets collected from mice after 18 days of colonization were homogenized in sterile saline and plated on YPD-SA to quantify fungal load. No CFU were detected in fecal pellets from uncolonized mice. Figure shows CFU per gram from the C. albicans*-*colonized mice. Each symbol represents a mouse, and each bar indicates the average. Download FIG S1, TIF file, 0.2 MB. Copyright © 2021 Markey et al. 2021 Markey et al. This content is distributed under the terms of the Creative Commons Attribution 4.0 International license.

C. albicans colonization had a modest but statistically significant impact on the diversity and composition of the bacterial microbiota. Fecal pellets were collected prior to clindamycin challenge, and bacterial DNA was extracted from fecal pellets. We used quantitative PCR (qPCR) to measure the total amount of bacterial DNA present before clindamycin challenge (day −1 in Fig. 1A) and observed a statistically significant but small increase in total bacterial abundance in the C. albicans-colonized mice (Fig. 1B) (*P* = 0.0065, *t* test).

Standard 16S rRNA DNA sequencing using Illumina MiSeq and analysis using QIIME2 was performed on the fecal pellet DNA samples. At the phylum level, little difference was observed in the relative abundance of phyla between the microbiota of C. albicans*-*colonized versus uncolonized mice (see Fig. S2A). No statistically significant differences in phylum level relative abundance, corrected for multiple comparisons, were detected.

10.1128/mSphere.00982-20.3 FIG S2 Relative abundances of bacterial phyla and all bacterial genera with a median greater than zero. Mice were pretreated with cefoperazone for 10 days, colonized with C. albicans or not, and switched to sterile water for 3 weeks, during which time the bacterial microbiota recovered. Fecal pellets were collected at the end of the recovery period prior to clindamycin treatment, and the microbiota were analyzed by 16S rRNA sequencing and QIIME2. (A) The proportion each phylum makes up of the total bacterial microbiota population, or relative abundance, in uncolonized microbiota or C. albicans-colonized microbiota. Bars show the means with the SEMs. (B) The proportion each genus makes up of the total bacterial microbiota population, or relative abundance. Bars indicate averages and SEMs. Those genera identified as significantly different in abundance by LEfSe are highlighted in the red box. Uncolonized, *N* = 20; C. albicans colonized, *N* = 24. Abbreviations: Corio, unclassified *Coriobacteriaceae*; Turic, *Turicibacter*; Bifido, *Bifidobacterium*; Bact, *Bacteroides*; Staph, *Staphylococcus*; Lacto, *Lactobacillus*; Un. Clostr, unclassified *Clostridiales*; Christ, unclassified *Christensenellaceae*; Clost, *Clostridium*; Dehalo, *Dehalobacterium*; Un. Lachno, unclassified *Lachnospiraceae*; Anaeros, *Anaerostipes*; Copro, *Coprococcus*; Rumino, *Ruminococcus*; Un. Rumino, unclassified *Ruminococcaceae*; Butyr, *Butyricicoccus*; Oscill, *Oscillospira*; Rumino, *Ruminococcaceae Ruminococcus*; Un. Mogi, unclassified *Mogibacteriaceae*; Un. Erysip, unclassified *Erysipelotrichaceae*; Allob, *Allobaculum*; E. Clostri, *Erysipelotrichaceae Clostridium*; Sutt, *Sutterella*; Anaerop, *Anaeroplasma*; Akk, *Akkermansia*. Download FIG S2, TIF file, 0.9 MB. Copyright © 2021 Markey et al. 2021 Markey et al. This content is distributed under the terms of the Creative Commons Attribution 4.0 International license.

Analysis of the relative composition of the microbiota at the genus level revealed some differences that accompanied C. albicans colonization (Fig. S2B). Relative abundance of all genera with a median of >0 in at least one group are shown in Fig. S2B. Most of the genera were present at a similar relative abundance in microbiota with or without C. albicans. An expansion of *Akkermansia* and a decrease in *Bacteroides* in the C. albicans-colonized mice were observed. We also used linear discriminant analysis effect size (LEfSe) (26) to further investigate differences in relative abundance of various taxonomic groups, considering taxa with a median of >0 in at least one group, and determined that six genera were significantly different in abundance between the C. albicans-colonized and uncolonized groups (Fig. 1C; and shown in red box in Fig. S2B), including the most abundant genus in both populations, *Bacteroides* (Fig. 1C) (*P* = 0.022, *t* test, fold change of 0.71). These results demonstrate that the majority of bacterial genera were found at similar levels in the microbiota of uncolonized and C. albicans*-*colonized mice.

Factors that affect microbiota composition often have a strong effect and produce changes in composition that can be detected at the phylum level. For example, dietary changes (27, 28) and colonization with diverse microbes (e.g., Cryptosporidium parvum [29], Vibrio parahaemolyticus [30], and Klebsiella pneumoniae [31]) produce phylum-level changes. In contrast, the compositions of uncolonized and C. albicans-colonized mice under the conditions of our experiments showed minimal differences at the phylum level (Fig. S2A), with some differences detected at the genus level (Fig. S2B). Based on these comparisons, we consider the differences between uncolonized and C. albicans-colonized mouse fecal microbiota to be modest and describe these communities as being similar.

Alpha diversity analysis of the pre-clindamycin community (not excluding genera with median = 0) determined that the microbiota of C. albicans-colonized mice was less diverse than that of the uncolonized mice as measured by the Simpson index (Fig. 1D) (*P* = 0.0089, Welch’s *t* test). Differences were also detected with some additional metrics of alpha diversity (see Fig. S3). Principal-coordinate analysis of the weighted UniFrac distance matrix including all pre-clindamycin samples showed a trend toward separation of the C. albicans-colonized and uncolonized populations (Fig. 1E) (permutational multivariate analysis of variance [PERMANOVA] *P* = 0.055, 999 permutations). Together, these results indicated that colonization with C. albicans had modest effects on the composition and diversity of the bacterial gut microbiota. Previous studies of the effect of C. albicans colonization on the bacterial microbiota using a cefoperazone-treated mouse model are in agreement with our finding of detectable but modest changes (14, 20), although the identities of the genera affected differed between studies, likely due to differences in starting microbiota compositions. Effects of C. albicans colonization on bacterial abundance and diversity in the human gut (32) and in the oral cavities of immunocompromised mice (33, 34) were also previously identified.

10.1128/mSphere.00982-20.4 FIG S3 Alpha diversity of the pre-clindamycin microbiota. Mice were pretreated with cefoperazone in drinking water for 10 days and then either colonized with C. albicans (5 × 10^7^ CFU by oral inoculation) or not. Mice were then switched back to sterile water, and the microbiota were allowed to recover for 3 weeks. The fecal pellet microbiota was analyzed with 16S rRNA sequencing and the QIIME2 analysis pipeline. (A to D) Alpha diversity metrics. Symbols indicate individual mouse microbiota diversity and bars indicate the averages and SEMs. ●, uncolonized mouse microbiota (*N *= 20); △, C. albicans-colonized microbiota (*N* = 24). Welch’s unpaired *t* test was used. Download FIG S3, TIF file, 0.4 MB. Copyright © 2021 Markey et al. 2021 Markey et al. This content is distributed under the terms of the Creative Commons Attribution 4.0 International license.

As a novel microbe colonizing the GI tract, C. albicans could have affected the bacterial microbiota through multiple physiological pathways. Previous researchers have characterized multiple changes in the immune environment of the gut in response to C. albicans colonization (35, 36), and Romo et al. demonstrated that C. albicans alters the metabolite milieu of the gut through increased abundance of nonesterified unsaturated fatty acids and other lipid species (37). In sum, our study adds to the body of work demonstrating that C. albicans, a fungus, mildly alters the composition of the bacterial microbiota in the context of the mouse GI tract environment.

### Network analysis of the C. albicans*-*colonized microbiota showed decreased centrality and decreased modularity compared to those of the uncolonized microbiota.

**FIG 2:**
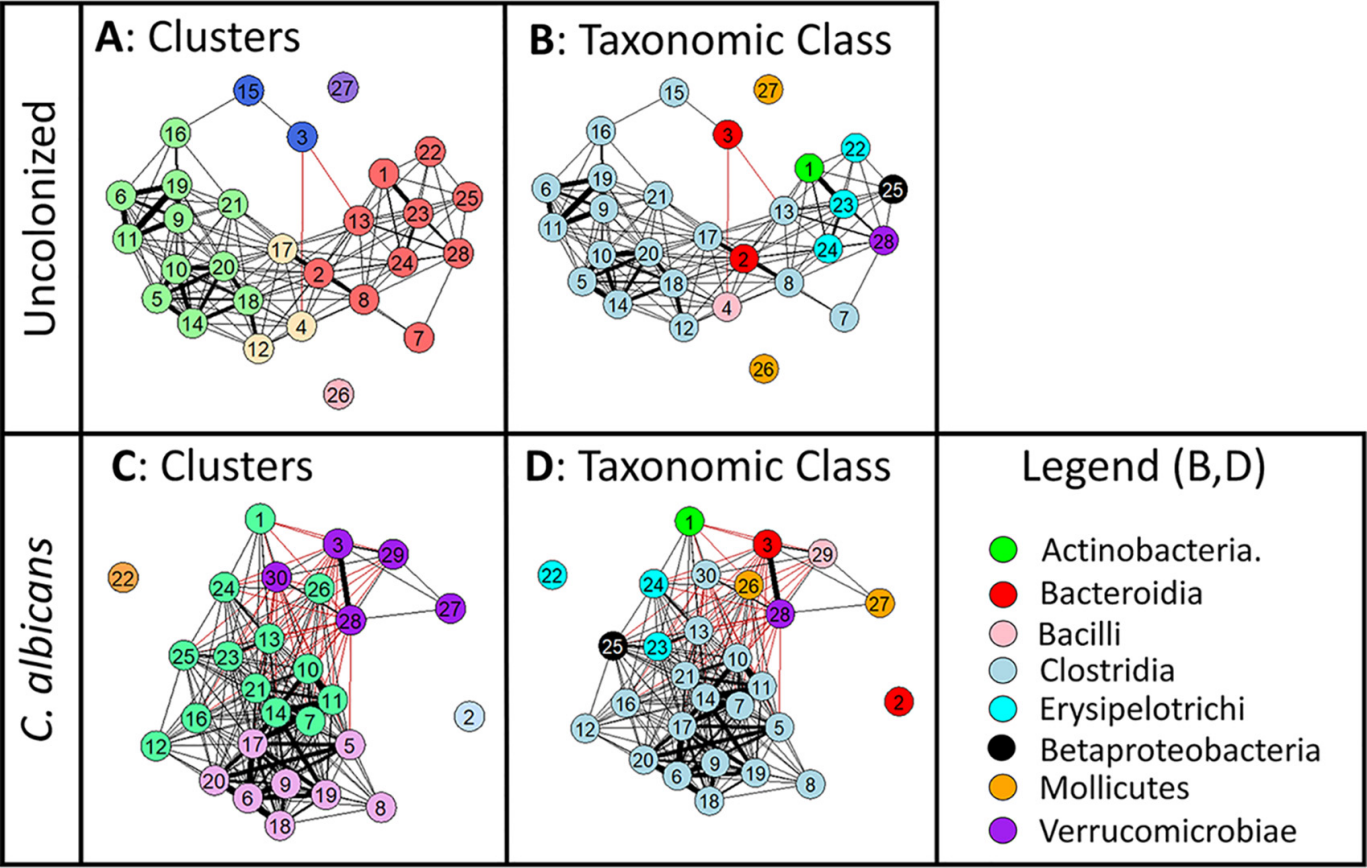
C. albicans colonization altered network topology of the bacterial microbiota. Mice were pretreated with cefoperazone in drinking water for 10 days and then either colonized with C. albicans (5 × 10^7^ CFU by oral inoculation) or not. Mice were then switched back to sterile water, and the microbiota were allowed to recover for 3 weeks. 16S rRNA sequencing was used to analyze the bacterial microbiota of fecal pellets collected prior to clindamycin treatment (day −1). The sequencing data were analyzed using QIIME2 and taxonomic identification of amplicon sequence variants (ASVs) at the genus level performed by matching to the Greengenes database. Absolute abundance of each genus was determined by multiplying the relative abundance of that genus by the total quantity of bacterial DNA normalized to grams of fecal pellet material measured by qPCR using universal primers. The absolute abundance of each genus was correlated with the abundance of every other genus using Pearson correlation, and this correlation matrix was used for network analysis (A to D) using qgraph. Each node represents a genus (described in Table 1), and each edge and its thickness represent the strength (*R*) of the correlation between those nodes. Only statistically significant edges are shown, *P* < 0.05 after Benjamini-Hochberg correction. Positive correlations are shown as black edges and negative correlations are red edges. (A and B) Co-occurrence network of the uncolonized bacterial microbiota. (C and D) C. albicans-colonized microbiota network. Nodes are colored by cluster (A and C) as determined by fast-greedy cluster analysis using igraph, such that different colors indicate separate community clusters, and by taxonomic class (B and D), for which the legend is shown far right. Network analysis of the pre-clindamycin microbiota included mice from two cohorts: total uncolonized, *N* = 20; C. albicans colonized, *N* = 24.

Previous research has demonstrated that the bacterial gut microbiota exhibits network effects (38) and that network structure can affect the stability of the microbiota community over time and in response to perturbation (21). To supplement the analysis of the effects of C. albicans colonization on bacterial microbiota composition shown above, we used network analysis of the pre-clindamycin microbiota (day −1, Fig. 1A) to gain insight into the relationships between genera and determine how these connections might be differentially affected by C. albicans colonization. Co-occurrence network analysis was conducted using microbiota sequencing data from a total of 20 uncolonized mice and 24 C. albicans-colonized mice from two experimental trials. The absolute abundance (relative abundance measured by sequencing [Fig. S2B] multiplied by the total bacterial abundance measured by qPCR per gram of fecal material [Fig. 1B]) of each genus was used for this analysis, and only genera with a median abundance greater than zero were included. The abundance of each genus was correlated with the abundance of every other genus using Pearson’s correlation, and the resulting correlation matrix (see Fig. S4) was used as the input for network analysis with igraph (39) and qgraph (40) within R. Despite significant differences in starting compositions between experimental trials (see Fig. S5), there were sufficient statistically significant correlations between genera to generate a co-occurrence network for bacterial genera comprising the uncolonized and C. albicans-colonized bacterial microbiota (Fig. 2). Each numbered node represents a genus, and connecting edges indicate statistically significant correlations (*P* < 0.05, Benjamini-Hochberg correction for multiple comparisons). Genera included in the analysis and their absolute abundance are summarized in Table 1. Layout of nodes within the network was determined using the force-directed Fruchterman-Reingold algorithm (41) with the repulsion parameter set to 0.75 to limit dispersion, such that highly correlated nodes are pulled closer together and uncorrelated nodes are pushed farther apart.

**TABLE 1:** Genera included in network analysis of the pre-clindamycin mouse fecal microbiota

No.^*a*^	Genus^*b*^	Class^*b*^	Abundance^*c*^		Node centrality^*d*^	
			Uncolonized	C. albicans colonized	Uncolonized	C. albicans colonized
1	*Bifidobacterium*	*Actinobacteria*	4.71 × 10^3^	1.14 × 10^4^	8.34	5.20
2	*Bacteroides*	*Bacteroidia*	1.59 × 10^5^	1.57 × 10^5^	14.6	−1.78
3	Unclassified *S24-7*	*Bacteroidia*	3.28 × 10^4^	6.50 × 10^4^	−5.22	−5.16
4	*Staphylococcus*	*Bacilli*	8.96 × 10^1^	Median = 0	10.0	Median = 0
5	Unclassified *Clostridiales*	*Clostridia*	6.81 × 10^3^	1.20 × 10^4^	13.0	10.7
6	Unclassified *Clostridiales*	*Clostridia*	1.02 × 10^5^	1.23 × 10^5^	10.5	12.4
7	Unclassified *Christensenellaceae*	*Clostridia*	3.21 × 10^2^	6.79 × 10^2^	9.92	11.4
8	*Clostridium* (*Clostridiaceae*)	*Clostridia*	1.43 × 10^3^	9.36 × 10^2^	13.6	9.22
9	*Dehalobacterium*	*Clostridia*	8.89 × 10^2^	9.95 × 10^2^	11.2	11.9
10	Unclassified *Lachnospiraceae*	*Clostridia*	4.73 × 10^4^	4.25 × 10^4^	13.8	11.4
11	Unclassified *Lachnospiraceae*	*Clostridia*	1.96 × 10^4^	4.53 × 10^4^	11.3	12.2
12	*Coprococcus*	*Clostridia*	6.22 × 10^3^	2.87 × 10^3^	12.1	8.56
13	*Dorea*	*Clostridia*	6.60 × 10^3^	7.16 × 10^3^	11.4	10.7
14	*Ruminococcus* (*Lachnospiraceae*)	*Clostridia*	1.35 × 10^4^	1.66 × 10^4^	13.0	12.5
15	*rc4-4*	*Clostridia*	1.83 × 10^3^	Median = 0	0.380	Median = 0
16	Unclassified *Ruminococcaceae*	*Clostridia*	2.84 × 10^2^	1.03 × 10^3^	6.94	10.2
17	Unclassified *Ruminococcaceae*	*Clostridia*	6.42 × 10^3^	1.03 × 10^4^	14.7	13.1
18	*Butyricicoccus*	*Clostridia*	1.06 × 10^3^	1.44 × 10^3^	14.0	11.5
19	*Oscillospira*	*Clostridia*	2.87 × 10^4^	3.77 × 10^4^	11.0	11.6
20	*Ruminococcus* (*Ruminococcaceae*)	*Clostridia*	1.04 × 10^4^	1.11 × 10^4^	14.8	11.4
21	Unclassified *Mogibacteriaceae*	*Clostridia*	4.17 × 10^2^	1.17 × 10^3^	12.7	12.0
22	Unclassified *Erysipelotrichaceae*	*Erysipelotrichia*	6.18 × 10^2^	5.68 × 10^2^	8.13	0.441
23	*Allobaculum*	*Erysipelotrichia*	3.34 × 10^4^	5.89 × 10^4^	9.25	10.8
24	*Clostridium* (*Erysipelotrichaceae*)	*Erysipelotrichia*	2.03 × 10^3^	1.94 × 10^3^	9.43	8.15
25	*Sutterella*	*Betaproteobacteria*	5.29 × 10^3^	1.00 × 10^4^	7.77	9.12
26	*Anaeroplasma*	*Mollicutes*	1.07 ×10^3^	4.84 × 10^3^	3.43	6.31
27	Unclassified *RF39*	*Mollicutes*	1.88 × 10^2^	4.29 × 10^2^	3.83	−3.55
28	*Akkermansia*	*Verrucomicrobiae*	7.94 × 10^4^	2.04 × 10^5^	7.97	−8.37
29	*Lactobacillus*	*Bacilli*	Median = 0	8.91 × 10^3^	Median = 0	−4.60
30	*Anaerostipes*	*Clostridia*	Median = 0	1.72 × 10^3^	Median = 0	−8.46

a Numbers indicate the numbers used to label nodes in Fig. 2 and 4.

b Taxonomic information about genera included in the analysis. Distinct ASVs that were identified as the same genus remain distinct nodes (i.e., nodes 5 and 6).

c Average absolute abundance of the genus (i.e., relative abundance × total bacterial DNA per gram fecal pellet measured by qPCR).

d Measure of node centrality, the “expected influence” metric that calculates the importance of a node to the network as a whole based on the number and strength of the edges connecting that node to other nodes.

10.1128/mSphere.00982-20.5 FIG S4 Correlation matrix of the pre-clindamycin microbiota in uncolonized and C. albicans-colonized mice. Mice were pretreated with cefoperazone for 10 days, colonized with C. albicans or not, and switched to sterile water for 3 weeks, during which time the bacterial microbiota recovered. Fecal pellets were collected at the end of the recovery period prior to clindamycin treatment, and the microbiota were analyzed by 16S rRNA sequencing and QIIME2. This figure summarizes the Pearson correlations between the absolute abundance of each genus with every other genus in the uncolonized and C. albicans-colonized microbiota. Only statistically significant correlations are shown. The larger and darker the square within the grid, the more significant the correlation. The color corresponds to the value of *R*, shown on the right. Numbers refer to genera listed in Tables 1 and 2. Only genera with a median abundance greater than zero were included in the analysis. Download FIG S4, TIF file, 1.7 MB. Copyright © 2021 Markey et al. 2021 Markey et al. This content is distributed under the terms of the Creative Commons Attribution 4.0 International license.

10.1128/mSphere.00982-20.6 FIG S5 Significant differences in beta diversity between experimental cohorts. Mice were pretreated with cefoperazone for 10 days and then the bacterial microbiota were allowed to recover for 3 weeks. Fecal pellets were collected at the end of this period and were used for 16S rRNA analysis of the bacterial microbiota (pre-clindamycin). Each symbol represents an individual mouse microbiota, and shapes indicate different experimental cohorts. Separation between the two cohorts was statistically significant as measured by PERMANOVA, 999 permutations, pseudo-*F* = 26.53, *P* = 0.001. Download FIG S5, TIF file, 0.2 MB. Copyright © 2021 Markey et al. 2021 Markey et al. This content is distributed under the terms of the Creative Commons Attribution 4.0 International license.

In the uncolonized microbiota, community analysis of the microbiota network using a fast-greedy clustering algorithm (42) identified three distinct clusters (as well as additional genera not significantly correlated with other genera), shown as different colored nodes in Fig. 2A. A subgroup exclusively consisting of the taxonomic class *Clostridia* is part of one highly correlated cluster, while the dominant genus *Bacteroides* (numbered 2) was a part of a separate cluster of highly correlated genera which included members of five different taxonomic classes (Fig. 2B). The two genera that were not connected to any other genus (26 and 27) were both members of the class *Mollicutes* and were the only members of that class detected with a median abundance greater than zero, indicating that this class of bacteria, in general, was not integrated into the microbiota network of the uncolonized mice.

C. albicans colonization resulted in significant topological changes to the bacterial microbiota co-occurrence network. Abundance of C. albicans was not included in the co-occurrence network analysis, as C. albicans is a fungus. Two genera (*Staphylococcus* and *rc4-4*) that were included in the uncolonized microbiota network analysis were omitted from the C. albicans-colonized analysis because their median abundance was not greater than zero. Conversely, the C. albicans-colonized network included two genera (*Lactobacillus* and *Anaerostipes*) that were not included in the analysis of the uncolonized microbiota.

The network of correlations in the C. albicans-colonized microbiota retained many of the statistically significant positive correlations observed in the uncolonized mice and included multiple negative correlations not observed in the uncolonized microbiota network (Fig. S4). As for the uncolonized microbiota network, community clustering analysis revealed statistically significant clusters in the C. albicans-colonized network that included both taxonomically distinct clusters (one exclusively *Clostridia*) and clusters including genera from multiple taxonomic classes (Fig. 2C and D). The numbers and strengths of correlations were more evenly distributed between nodes in the C. albicans-colonized network, resulting in a decrease in overall network centrality and modularity compared to that for the uncolonized network (Table 2) and increased network density with a shorter average path length between any two nodes. These changes in network topology metrics reflect a general change in the structure of the network, from the distinct modules centered around a few highly influential nodes observed in the uncolonized microbiota to a highly connected network in which node influence is more evenly distributed between all genera in the C. albicans-colonized network.

**TABLE 2:** Total network topography

Parameter^*a*^	Value	
	Uncolonized	C. albicans colonized
Avg path length (no. of edges)	1.91	1.20
Density	0.36	0.47
Linkage density	4.96	7.96
Connectance	0.37	0.59
Overall centrality	0.10	0.01
Overall modularity	0.28	0.13

a igraph was used to measure standard measures of network topography of the uncolonized and C. albicans-colonized pre-clindamycin networks shown in Fig. 2 and 4. The parameters (except for average path length) are ratios.

This overall decrease in centrality in the C. albicans-colonized versus that for the uncolonized networks is reflected in changes in one-step expected influence (43), one measure of degree centrality, for several notable genera including *Akkermansia* (Fig. 2, numbered 28; Table 1, right columns). The value of expected influence is the sum of all of the edges that connect one node to another, in which edges retain their sign. As the value of edges indicates the strength of correlations, the number, sign, and strength of correlations all contribute to the expected influence of a node. We observed a −16 change in expected influence for *Akkermansia*, as it was positively correlated with seven genera in the uncolonized microbiota but highly negatively correlated with 12 genera in the C. albicans-colonized microbiota. This change in influence indicated that *Akkermansia* remained central to the C. albicans-colonized network but that its interactions with other genera had shifted significantly.

Using DiffCorr (44) and Fisher’s Z-test in R, we identified 39 statistically significant differences in correlation values between the uncolonized and C. albicans-colonized microbiotas (see Table S1). Of the 10 significantly different pairwise correlations with the lowest *P* values (Table 3), seven pairs included *Akkermansia*, implying that C. albicans colonization had strong effects on the correlations and, potentially, interactions between *Akkermansia* and other bacterial genera. While *Akkermansia* was significantly correlated with the genera listed in both the uncolonized and C. albicans-colonized microbiota, the nature of the correlation switched from being highly positively correlated to negatively correlated or vice versa (Table 3). Compositional analysis (Fig. 1C; Fig. S2B) did not demonstrate a significant effect of C. albicans on the abundance of the genera identified by DiffCorr, implying that the change in correlation was not a result of an average change in bacterial abundance but rather the result of a change in the degree of co-occurrence of the pairs of bacterial genera in individual microbiota communities. Together, the DiffCorr analysis and analysis of changes in node centrality indicate that C. albicans colonization has a significant effect on the position of *Akkermansia* in the bacterial microbiota network.

**TABLE 3:** Differential correlations between genera in the C. albicans versus uncolonized networks

Node no.^*a*^	Genus^*b*^	Node no.^*a*^	Genus^*b*^	*R*^*c*^		*P* value of difference^*d*^
				Uncolonized	C. albicans colonized	
28	*Akkermansia*	23	*Allobaculum*	0.87	−0.71	1.55 × 10^−9^
28	*Akkermansia*	1	*Bifidobacterium*	0.80	−0.63	9.62 × 10^−7^
28	*Akkermansia*	13	*Dorea*	0.85	−0.76	1.55 × 10^−9^
28	*Akkermansia*	24	*Clostridium* (*Erysipelotrichaceae*)	0.78	−0.70	3.31 × 10^−7^
28	*Akkermansia*	7	Unclassified *Christensenellaceae*	0.58	−0.64	4.39 × 10^−4^
28	*Akkermansia*	22	Unclassified *Erysipelotrichaceae*	0.81	−0.27	5.01 × 10^−4^
28	*Akkermansia*	3	Unclassified *S24-7*	−0.50	0.92	5.99 × 10^−9^
8	*Clostridium* (*Clostridiaceae*)	2	*Bacteroides*	0.87	−0.12	3.71 × 10^−4^
19	*Oscillospira*	11	Unclassified *Lachnospiraceae*	0.997	0.82	2.62 × 10^−4^
17	Unclassified *Ruminococcaceae*	2	*Bacteroides*	0.91	−0.16	1.20 × 10^−5^

a Node number as shown in Fig. 2 and 4.

b Genus corresponding to the node number.

c DiffCorr was used to measure the differential correlation between shared genera in the C. albicans versus uncolonized pre-clindamycin networks shown in Fig. 2 and 4. The 10 pairs of genera with the most significant change in correlation are shown (full list is shown in Table S1 in the supplemental material).

d Differential correlation was calculated using Fisher’s Z-test with the Benjamini-Hochberg correction for multiple comparisons.

10.1128/mSphere.00982-20.1 TABLE S1 Significant changes in genus correlations. DiffCorr was used to compare the uncolonized and C. albicans correlation matrices (see Fig. S4). Fisher’s Z-test was used to determine whether correlation was significantly different between experimental groups. Those with a *P* value of <0.05 are shown (corrected for multiple comparisons using Benjamini-Hochberg method). Download Table S1, DOCX file, 0.02 MB. Copyright © 2021 Markey et al. 2021 Markey et al. This content is distributed under the terms of the Creative Commons Attribution 4.0 International license.

Analysis of node centrality revealed a striking change in the position of *Bacteroides* (Fig. 2, numbered 2) in the uncolonized versus C. albicans-colonized microbiota network. In the uncolonized microbiota network, *Bacteroides* had a high expected influence value as a highly centralized node with positive correlations connected to multiple clusters (Fig. 2A and B; Table 1). However, in the C. albicans-colonized microbiota, *Bacteroides* had no significant correlations with any other genera and was no longer a member of a centralized community cluster, resulting in a change in expected influence of −16. This change in implied community interaction was also reflected in the DiffCorr analysis, which highlighted the significant difference in correlation between *Bacteroides* and *Clostridiaceae Clostridium* or between *Bacteroides* and unclassified *Ruminococcaceae* in the uncolonized and C. albicans-colonized microbiota. *Bacteroides* remained abundant in the C. albicans*-*colonized microbiota, although there was a small but significant (0.7-fold) decrease in *Bacteroides* abundance (Fig. 1C) that may have contributed to the decreased integration of *Bacteroides* into the C. albicans-colonized microbiota network. Finally, there was a significant decrease in correlation between *Oscillospira* and unclassified *Lachnospiraceae* in the C. albicans-colonized microbiota compared to that in the uncolonized microbiota, neither of which were significantly altered in abundance in the C. albicans-colonized mice. These observations provided additional evidence that C. albicans colonization altered the structure of the microbiota network without affecting the abundance of the majority of bacterial genera.

The exclusion of *Bacteroides* from the microbiota network and the shifting position of *Akkermansia* as a consequence of C. albicans colonization are particularly interesting because colonization with these common human commensal bacteria has been shown to have a positive impact on host health. Higher *Bacteroides* abundance has been correlated with reduced susceptibility to Clostridioides difficile disease in human patients (45) and reduced susceptibility to enteric pathogens Enterococcus faecium (46) and Salmonella enterica (47) in mice. Colonization with Akkermansia muciniphila improves metabolic markers in mice with genetic or diet-induced obesity (48, 49). A decreased abundance of *A. muciniphila* is correlated with metabolic disorder in human cohorts (50, 51) and in mouse models (52). The shifts in network positions of *Bacteroides* and *Akkermansia* as a consequence of C. albicans colonization suggest that the addition of C. albicans can alter microbiota dynamics of these clinically relevant commensal bacterial genera, even while these genera remain abundant members of the microbiota.

More broadly, changes in the network structure of the microbiota could have a significant impact on how a host responds to subsequent perturbations such as antibiotic treatment or infection with a pathogen. For example, Xiao et al. demonstrated that bacterial microbiota network structure could be used to predict FMT efficacy for treatment of recurrent infection by Clostridioides difficile by comparing the microbiota networks of donors and recipients (38). In the next set of experiments, we examined whether the differences in microbiota composition and network structure of uncolonized or C. albicans*-*colonized mice affected the ecological resistance of the bacterial communities.

### C. albicans colonization decreased ecological resistance of the bacterial microbiota in a clindamycin challenge model.

The goal of the next series of experiments was to determine whether differences in composition, diversity, and network structure between microbiota of uncolonized or C. albicans*-*colonized mice had functional significance. We chose to measure the ecological resistance of the community to challenge with an antibiotic, clindamycin, to detect the ability of the microbiota to resist perturbation with this antibiotic.

To this end, mice were challenged with a range of concentrations of clindamycin in order to probe the response of the bacterial microbiota ecosystem to a broadly disruptive intervention. After 3 weeks of bacterial microbiota recovery post-C. albicans inoculation (Fig. 1A), mice were treated with a low (1.11 mg/kg body weight), intermediate (3.33 mg/kg), or high (10 mg/kg) dose of clindamycin to determine the effect of C. albicans colonization on the response of the microbiota to disruption. Each mouse was treated with clindamycin only one time, by intraperitoneal injection. Fecal pellets were collected for bacterial microbiota analysis 1 day prior to clindamycin treatment (day −1, Fig. 1A) and 1 day after clindamycin treatment (day 1, Fig. 1A).

**FIG 3:**
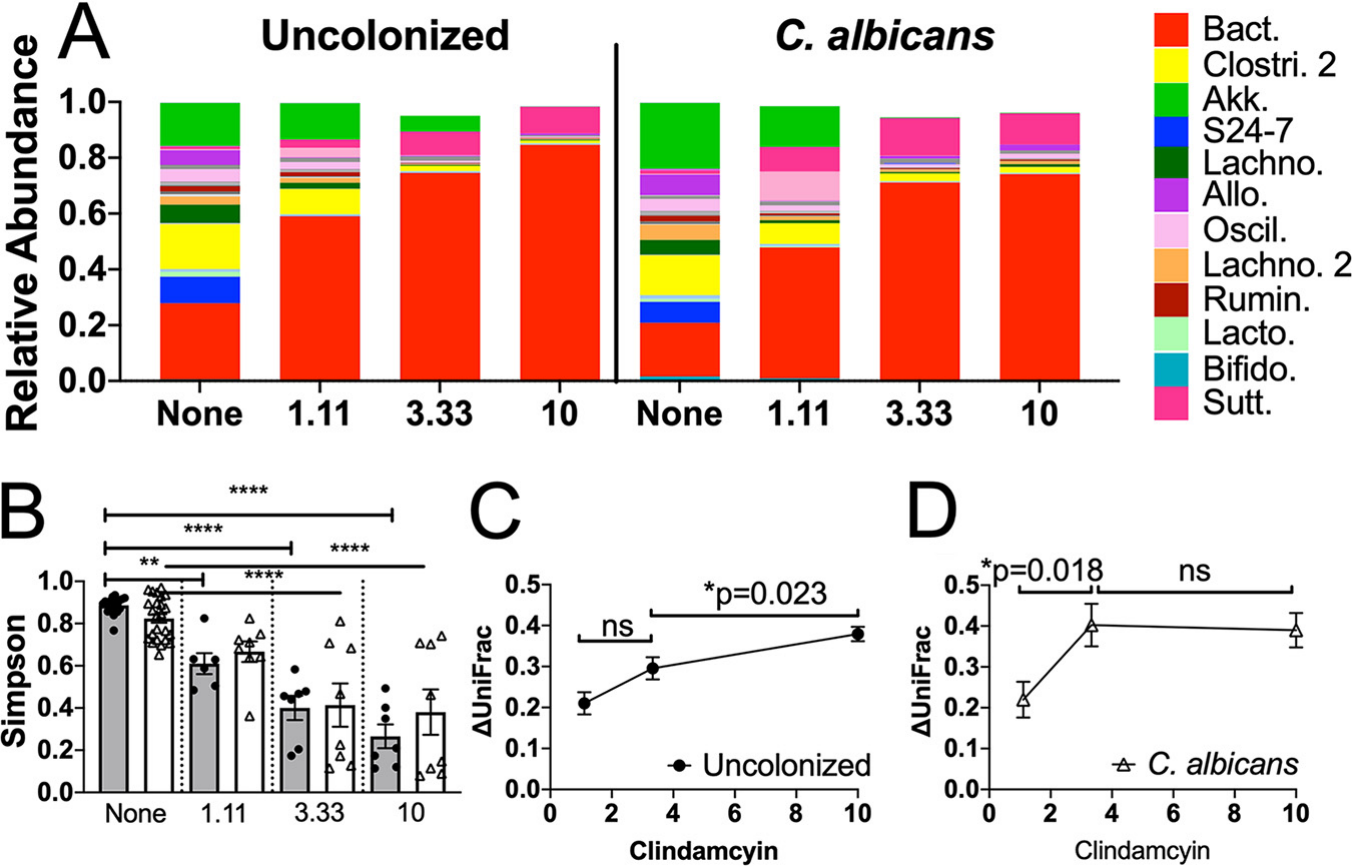
C. albicans colonization decreased ecological resistance of the bacterial microbiota. Mice were pretreated with cefoperazone in drinking water for 10 days and then either colonized with C. albicans (5 × 10^7^ CFU by oral inoculation) or not. Mice were then switched back to sterile water, and the microbiota were allowed to recover for 3 weeks. They then received a single intraperitoneal (i.p.) injection of 1.11 mg/kg body weight, 3.33 mg/kg, or 10 mg/kg clindamycin. Fecal pellets were collected 1 day before and 1 day after clindamycin treatment for bacterial microbiota analysis by 16S rRNA sequencing and QIIME2. (A) Relative abundances of all bacterial genera detected with a median of >0 pre-clindamycin, before and after clindamycin challenge, in the average uncolonized and C. albicans-colonized microbiota. The colored proportion of the bar represents the average proportion of the whole microbiota for each individual genus. The most abundant bacterial genera are shown in the legend at the right: Bact, *Bacteroides*; Clostr. 2, unclassified *Clostridiales* 2; Akk, *Akkermansia*; S24-7, unclassified S24-7; Lachno, unclassified *Lachnospiraceae*; Allo, *Allobaculum*; Oscil, *Oscillospira*; Lachno. 2, unclassified *Lachnospiraceae* 2; Rumin, unclassified *Ruminococcaceae*; Lacto, *Lactobacillus*; Bifido, *Bifidobacteria*; Sutt, *Sutterella*. (B) Simpson indices before and after clindamycin challenge in uncolonized and C. albicans-colonized mice. Symbols indicate individual mouse microbiota, bars indicate the averages, and error bars indicate SEMs. Gray bars/● indicate uncolonized mice and open bars/△ C. albicans colonized. ANOVA followed by Sidak’s *post hoc* test: **, *P* < 0.01; ****, *P* < 0.0001. Average changes in weighted UniFrac position from starting position of the uncolonized microbiota (C) and the C. albicans-colonized microbiota (D) as a consequence of clindamycin treatment, change in position calculated for each individual, and the average of experimental groups. Symbols indicate the averages and error bars indicate the SEMs. Welch’s *t* test was used to compare experimental groups; except where noted, *P* < 0.05. Uncolonized (C): 1.11 mg/kg, *N* = 6; 3.33 mg/kg, *N* = 7; 10 mg/kg, *N* = 7. C. albicans-colonized (D): 1.11 mg/kg, *N* = 8; 3.33 mg/kg, *N* = 8; 10 mg/kg, *N* = 8.

The compositions of genera in the fecal microbiota before and after treatment of mice with clindamycin are shown in Fig. 3A. Relative abundances of genera were markedly altered by the treatment. Microbiota composition and diversity were more strongly affected by clindamycin treatment than by C. albicans colonization. LEfSe analysis of the relative abundance of genera after clindamycin challenge showed that 2 of the genera that showed significant differences in relative abundance in the pre-clindamycin microbiota (*Coprococcus* and *Anaeroplasma*) between uncolonized and C. albicans-colonized mice were also differentially abundant after clindamycin treatment (see Fig. S6).

10.1128/mSphere.00982-20.7 FIG S6 Relative abundance of genera highlighted by LEfSe. Fecal pellets were collected on day 1 post-clindamycin and used for analysis of the bacterial microbiota by 16S rRNA sequencing with the QIIME2 analysis pipeline. LEfSe analysis of the relative abundance of genera after clindamycin treatment was used to identify bacterial genera significantly associated with the uncolonized or C. albicans-colonized microbiota across clindamycin conditions. Figure shows the relative abundance of these genera before and after clindamycin treatment, with the clindamycin condition indicated by the color of the bar and colonization condition indicated on the *x* axis. Pre-clindamycin abundance is represented by the open/white bar; 1.11 mg/kg body weight abundance is shown in gray, 3.33 mg/kg abundance is represented by the striped bar, and 10 mg/kg abundance is shown in black. Bars indicate the averages and SEMs. Download FIG S6, TIF file, 0.2 MB. Copyright © 2021 Markey et al. 2021 Markey et al. This content is distributed under the terms of the Creative Commons Attribution 4.0 International license.

Alpha diversity of the microbiota was analyzed using QIIME2. The average starting diversity as measured by Simpson index was lower in the C. albicans-colonized mice than in the uncolonized microbiota (Fig. 1D). In response to the low dose of clindamycin, alpha diversity of the uncolonized microbiota decreased (Fig. 3B) (Sidak’s *post hoc* test, *P* = 0.0021), and that of the C. albicans-colonized microbiota did not change significantly (Fig. 3B) (Sidak’s *post hoc* test, *P* = 0.11), such that both groups were comparable after low-dose challenge (Fig. 3B) (Sidak’s *post hoc* test, *P* = 0.9929). In response to the intermediate or high dose of clindamycin, both the uncolonized and C. albicans-colonized microbiota decreased in diversity, which were significantly lower than their respective starting Simpson index alpha diversity values (Fig. 3B) (analysis of variance [ANOVA] followed by Sidak’s multiple-comparison test, *P* < 0.0001). The reduced Simpson index diversity after intermediate- or high-dose clindamycin challenge was comparable between the C. albicans-colonized and uncolonized microbiota. Analysis using other diversity metrics yielded similar results (see Fig. S7).

10.1128/mSphere.00982-20.8 FIG S7 Alpha diversity after clindamycin treatment. Fecal pellets were collected on day 1 post-clindamycin and used for analysis of the bacterial microbiota by 16S rRNA sequencing with the QIIME2 analysis pipeline. (A to D) Different diversity metrics in mice treated with a low (1.11 mg/kg), intermediate (3.33 mg/kg), or high (10 mg/kg) dose of clindamycin. Each symbol represents diversity of an individual mouse microbiota, and bars indicate the averages and SEMs. Uncolonized mouse microbiota (*N* = 20); C. albicans-colonized mouse microbiota (*N* = 24). Two-way ANOVA was used to assess the effect of C. albicans colonization and clindamycin treatment on alpha diversity, *P* < 0.05. Clindamycin had a significant effect on alpha diversity across all metrics: (A) OTUs, *P* = 0.0011; (B) Chao1, *P* = 0.0011; (C) Shannon, *P* = 0.0007; (D) Pielou’s evenness, *P* = 0.0009; C. albicans-colonization did not. Sidak’s *post hoc* test was used to compare alpha diversity of uncolonized and C. albicans*-*colonized microbiota within each clindamycin condition, and there were no significant differences. Download FIG S7, TIF file, 0.3 MB. Copyright © 2021 Markey et al. 2021 Markey et al. This content is distributed under the terms of the Creative Commons Attribution 4.0 International license.

Comparison of beta diversity based on the weighted UniFrac matrix in microbiota of mice with and without C. albicans following each dose of clindamycin did not detect significant differences based on colonization status (see Fig. S8). However, analysis of the extent of changes in beta diversity as a result of clindamycin treatment revealed differences.

10.1128/mSphere.00982-20.9 FIG S8 Beta diversity after clindamycin treatment. Fecal pellets were collected on day 1 post-clindamycin and used for analysis of the bacterial microbiota by 16S rRNA sequencing with the QIIME2 analysis pipeline. Unweighted UniFrac values were used as input for a distance matrix, and principal-coordinate analysis was used to compare uncolonized and C. albicans-colonized microbiota after treatment with 1.11 mg/kg, 3.33 mg/kg, or 10 mg/kg of clindamycin. Symbols indicate individual mouse microbiota; ●, uncolonized microbiota; △, C. albicans-colonized microbiota. PERMANOVA demonstrated no significant separation between uncolonized and C. albicans-colonized microbiota in any of the clindamycin conditions (999 permutations). Download FIG S8, TIF file, 0.2 MB. Copyright © 2021 Markey et al. 2021 Markey et al. This content is distributed under the terms of the Creative Commons Attribution 4.0 International license.

C. albicans colonization resulted in greater shifts in beta diversity in response to clindamycin challenge than observed in uncolonized mice. For uncolonized mice, the microbiota changed the least in response to the low dose of clindamycin; the intermediate dose changed the microbiota significantly less than the high dose (Fig. 3C) (*P* = 0.023, unpaired *t* test).

In contrast, the change in UniFrac position of the C. albicans-colonized microbiota exhibited a strong threshold effect at the intermediate dose of clindamycin, in that the low dose changed significantly less than the intermediate dose (Fig. 3D) (*P* = 0.018, unpaired *t* test) and the intermediate and high doses were comparable. The difference in the shapes of the responses (Fig. 3C versus Fig. 3D) implied a functional difference in how the microbiota responded to clindamycin treatment when C. albicans was present versus when it was absent. The C. albicans-colonized microbiota exhibited greater change in beta diversity in response to the intermediate dose of clindamycin, indicating that the microbiota as a whole was less resistant to change and thus had decreased ecological resistance.

**FIG 4:**
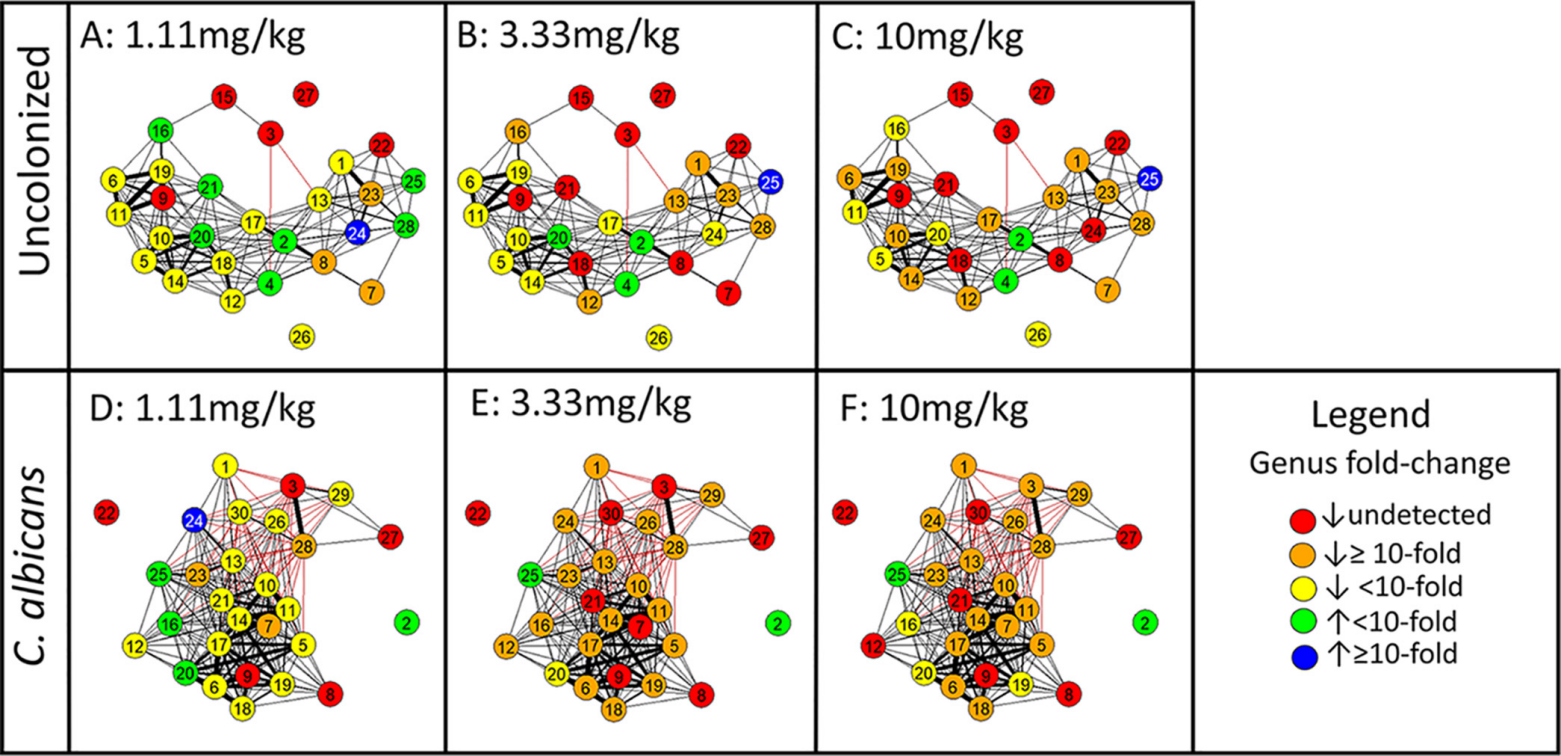
Clindamycin challenge has a differential effect on uncolonized and C. albicans-colonized microbiota networks. Network analysis was performed as described for Fig. 2. Only the pre-clindamycin network is shown, with nodes colored according to post-clindamycin response. (A to C) Co-occurrence network of the uncolonized bacterial microbiota. (D to F) C. albicans*-*colonized microbiota network. Nodes are colored by their response in fold change in absolute abundance after challenge with low-dose clindamycin (1.11 mg/kg), intermediate-dose clindamycin (3.33 mg/kg), or high-dose clindamycin (10 mg/kg). The legend is shown at the bottom right. Uncolonized (*N* = 20 total): 1.11 mg/kg, *N* = 6; 3.33 mg/kg, *N* = 7; 10 mg/kg, *N* = 7. C. albicans colonized (*N* = 24 total): 1.11 mg/kg, *N* = 8; 3.33 mg/kg, *N* = 8; 10 mg/kg, *N* = 8.

We examined the changes in individual genera more closely by coloring the pre-clindamycin co-occurrence network by the median fold change response of individual genera to low, intermediate, or high doses of clindamycin (Fig. 4A to F). After low-dose clindamycin challenge, the majority of genera in both the uncolonized microbiota and C. albicans-colonized networks appeared susceptible to clindamycin and decreased in abundance (20/28 [71%] and 23/28 [82%], respectively, indicated as yellow, orange, or red nodes), while inversely, 8/28 (29%, shown in green and blue nodes) increased in abundance in the uncolonized microbiota and 5/28 (18%, shown as green and blue nodes) increased in abundance in the C. albicans-colonized microbiota. In response to challenge with the intermediate dose of clindamycin, the majority of genera decreased in abundance in both the uncolonized and C. albicans*-*colonized microbiota (24/28 genera [86%] and 26/28 genera [93%], respectively). However, the fold change of the decrease was significantly greater in the C. albicans-colonized microbiota; in the uncolonized microbiota, only 15/24 genera (63%) that decreased in abundance did so by more than 10-fold, while in the C. albicans-colonized microbiota, 25/26 genera (96%) that decreased in abundance did so by more than 10-fold (*P* = 0.004, Fisher’s exact test). Thus, though the intermediate dose of clindamycin broadly decreased bacterial abundance across both the uncolonized and C. albicans-colonized networks, the C. albicans-colonized network again appeared less resistant to change at this dose, consistent with the change in beta diversity described above. At the high dose of clindamycin, both networks appeared comparably disrupted, as 73% of the genera in the uncolonized network and 72% of the genera in the C. albicans-colonized network decreased by 10-fold or more in abundance.

The four genera that increased after a high dose of clindamycin (*Bacteroides*, *Sutterella*, *Staphylococcus*, and unclassified *RF39*) differed from each other in their pre-clindamycin abundance, degree of influence, and taxonomic class; thus, the metrics measured in this analysis do not explain why these four genera are unaffected by clindamycin treatment. Clindamycin is a lincosamide antibiotic which is used to treat anaerobic, streptococcal, and staphylococcal infections, with *in vitro* bactericidal activity against a wide range of anaerobes, including Bacteroides fragilis as well as multiple *Staphylococcus* sp. (53, 54). However, clindamycin-resistant strains of both *Bacteroides* (55) and *Staphylococcus* (56) have been identified, and so it is possible that the strains present in this experiment were not sensitive to clindamycin.

Thus, the C. albicans-colonized microbiota appeared to be more affected by clindamycin at the intermediate dose than the uncolonized microbiota, as more genera were strongly decreased in abundance. These results reinforce the conclusions from analysis of beta diversity, which also indicated that the microbiota of C. albicans*-*colonized mice underwent a larger change in response to the intermediate and high doses of clindamycin than in response to the low dose. Treatment with the low dose of clindamycin had a small effect on the community, and treatment with the high dose had a strong enough effect that differences in community composition or network structure due to colonization status did not affect the response. At the intermediate dose, however, the milder effects of clindamycin treatment allowed us to observe colonization-associated differences in the response of the community to the antibiotic.

The differences in network structure described above, including increased node density, decreased modularity, and significant changes in correlations between key genera pairs, could contribute to the decreased ecological resistance of the C. albicans-colonized microbiota. Coyte et al., found that increasing cooperative interactions (inferred here by positive correlations in co-occurrence analysis) leads to interdependence and decreased community stability (21), in agreement with our observation that the more highly correlated C. albicans-colonized microbiota network was less stable in response to clindamycin challenge. Coyte et al. (21) also found that the introduction of spatial structure into the network, separating out clusters of nodes and weakening overall interactions, increased stability, which agrees with the increased modularity and cluster separation we observed in the more ecologically resistant uncolonized microbiota. Based on our findings, we suggest as a model that the relationships between bacterial genera inferred by the co-occurrence network analysis contribute to the community’s ecological resistance in response to clindamycin challenge and, further, that one effect of C. albicans colonization could be to alter the strength of these relationships. Therefore, structural changes in the microbiota network observed in C. albicans-colonized microbiota could result in the observed increase in sensitivity to disruption by clindamycin even though the compositions of the communities with and without C. albicans were very similar.

It is also possible that the decreased diversity observed in the C. albicans-colonized mice prior to clindamycin challenge contributed to the increased microbiota change in response to clindamycin challenge. Other researchers have shown that decreased diversity leads to decreased stability of the microbiota over time (57) and in response to subsequent antibiotic treatment, in agreement with our findings that colonization with C. albicans both decreased diversity and increased the amount of change in UniFrac position.

Furthermore, we note that the microbiota analysis considered bacteria at the genus level and, thus, would not detect changes at the species or strain level. Such changes could have occurred upon C. albicans colonization, producing communities that were more sensitive to clindamycin.

### Regardless of dose, bacterial microbiota recovers diversity by day five post-clindamycin treatment.

We conducted a longitudinal analysis of the bacterial microbiota to determine whether the microbiota recovered from the clindamycin challenge. Fecal pellets were collected on days −1, 1, 3, and 5 (Fig. 1A), and the bacterial microbiota was analyzed as described above. Alpha diversity over time was summarized using the Simpson metric. In the uncolonized mice, we observed a significant decrease across all doses on day 1 post-clindamycin treatment (as shown in Fig. 3B) followed by an increase in diversity on days 3 and 5 post-clindamycin (see Fig. S9A to C). On day 5, diversity was comparable to the pre-clindamycin level in mice treated with the low or intermediate dose of clindamycin but remained significantly reduced in the mice given the high dose of clindamycin (Fig. S9C) (two-way ANOVA followed by Dunnett’s *post hoc* test to compare post-clindamycin to day −1, *P* = 0.0045). In the C. albicans-colonized mice, there was no difference in diversity compared to that on day −1 on any day post-clindamycin in mice given the low dose (Fig. S9D) (two-way ANOVA followed by Dunnett’s *post hoc* test, *P* > 0.05). In C. albicans-colonized mice treated with the intermediate or high dose of clindamycin, we observed a decrease in diversity on day 1 post-clindamycin treatment (as shown in Fig. 3B) and an increase in diversity on days 3 and 5 post-clindamycin, such that on day 5 post-clindamycin, diversity in all groups was comparable to that on day −1 (Fig. S9D to F) (two-way ANOVA followed by Dunnett’s *post hoc* test, *P* > 0.05). This difference in diversity recovery between the uncolonized and C. albicans-colonized microbiota on day 5 relative to that pre-clindamycin likely reflects the decreased baseline diversity of the C. albicans-colonized microbiota pre-clindamycin rather than an increased ability of the C. albicans-colonized microbiota to recover after clindamycin challenge.

10.1128/mSphere.00982-20.10 FIG S9 Longitudinal analysis of alpha and beta diversity of the bacterial microbiota during the recovery from clindamycin treatment reveals dose- and C. albicans-dependent differences. Mice were given a single dose of clindamycin i.p. on day 0. Fecal pellets were collected prior to clindamycin (day −1) as well as on days 1, 3, and 5 after clindamycin, and the fecal pellet microbiota were sequenced and analyzed. (A to C) Average alpha diversities of the uncolonized bacterial microbiota as measured by the Simpson index are shown. (D to F) Average alpha diversities of the C. albicans*-*colonized bacterial microbiota as measured by the Simpson index are shown. Metrics were calculated for the microbiota of individual mice, and averages of experimental groups are shown. (A to F) Symbols indicate averages and bars indicate the SEMs. Two-way ANOVA followed by Dunnett’s *post hoc* test corrected for multiple comparisons was used to compare diversity at various timepoints to diversity on day −1, *P* < 0.05. (G to I) Average weighted UniFrac distances from the baseline are shown, where the baseline is the UniFrac position defined by beta diversity analysis of the microbiota from samples taken on day −1. Symbols indicate averages and bars indicate the SEMs. Two-way ANOVA was used to compare UniFrac distance from baseline and the effect of both C. albicans-colonization and timepoint post-clindamycin, *P* < 0.05. ●, uncolonized microbiota; △, C. albicans-colonized microbiota. Figures include data from two cohorts. Uncolonized: 1.11 mg/kg, *N* = 6; 3.33 mg/kg, *N* = 7; 10 mg/kg, *N* = 7. C. albicans-colonized: 1.11 mg/kg, *N* = 8; 3.33 mg/kg, *N* = 8; 10 mg/kg, *N* = 8. Download FIG S9, TIF file, 0.5 MB. Copyright © 2021 Markey et al. 2021 Markey et al. This content is distributed under the terms of the Creative Commons Attribution 4.0 International license.

The weighted UniFrac distance from the pre-clindamycin baseline was also measured. The low dose of clindamycin resulted in minimal change in UniFrac position for uncolonized or C. albicans-colonized mice (Fig. S9G). As on day 1 (Fig. 3C and D), the C. albicans-colonized microbiota treated with the intermediate dose had a greater change in UniFrac distance than the uncolonized microbiota on both days 3 and 5 (Fig. S9H) (two-way ANOVA, both time point and C. albicans-colonization had a significant effect, *P* = 0.0034 and *P* = 0.0004, respectively; there was no significant interaction between the two effects). The high dose of clindamycin resulted in greater changes in UniFrac distance on day 1 in both uncolonized and C. albicans-colonized microbiota, which decreased on days 3 and 5 post-clindamycin, indicating that during recovery from clindamycin, the microbiota shifted closer to the UniFrac position it occupied at baseline (Fig. S9I) (two-way ANOVA, time point had a significant effect, *P* = 0.0001; there was no significant effect of C. albicans-colonization or interaction between the two). Altogether this longitudinal analysis demonstrated that the bacterial microbiota of both uncolonized and C. albicans-colonized mice were sufficiently resilient to recover diversity by day five post-clindamycin.

In conclusion, the response of the microbiota to clindamycin challenge was used here to measure ecological resistance of the community. The introduction of C. albicans into the community had only modest effects on the composition of the bacterial microbiota. However, the underlying microbiota network structure was altered by colonization, and the C. albicans*-*containing community was more easily changed by clindamycin treatment. These studies provided insight into the microbiota response to acute clindamycin challenge and the effect of C. albicans colonization on ecological resistance.

## MATERIALS AND METHODS

### Strains and growth conditions.

C. albicans strain CKY101 (58), a virulent strain derived from the sequenced strain SC5314, was used for experiments which included C. albicans-colonized mice. For mouse inoculations, cells were grown at 37°C in YPD (1% yeast extract [BD 212750], 2% peptone [Difco 0118-17-0], 2% glucose [Sigma G8270]) (58) for 21 to 24 h, washed in phosphate-buffered saline (PBS), counted with a hemocytometer, and orally administered to animals as described below.

### Clindamycin challenge and GI colonization in mice.

All of the experiments included in this paper utilized 5-week-old female C57BL/6 mice (Jackson Laboratory). Two experimental trials were conducted, each using a cohort of mice shipped from the vendor. The two shipments resulted in a total of 20 uncolonized mice and 24 C. albicans-colonized mice whose fecal microbiota samples were sequenced. All mice from one shipment were co-housed in a large cage (24 in. by 17 in.) upon arrival at Tufts University. The mice were treated with antibiotic (cefoperazone [Sigma C4292], 0.5 g/liter) in drinking water for 10 days (Fig. 1A) while in the large cage. Mice were then divided into two large cages after cefoperazone treatment, such that one large cage contained all mice that would remain uncolonized and one large cage contained mice that were inoculated with C. albicans the following day. Prior to inoculation with C. albicans, mice were tested and shown to be negative for cultivable fungi by plating fecal pellet homogenates on YPD-SA agar medium (YPD agar plus 100 μg/ml streptomycin [Sigma S6501] and 50 μg/ml ampicillin [Sigma A9518]) and incubating for 2 days at 37°C. On the 10th day of antibiotic exposure, some mice were inoculated orally with 5 × 10^7^ C. albicans cells in 25 μl by gently pipetting into their mouths. All of the inoculated mice became colonized with C. albicans in the GI tract following a single inoculation. C. albicans colonization was measured over time by collecting fresh fecal pellets and plating homogenates on YPD-SA agar. Uninoculated mice in these studies were periodically confirmed as uncolonized by collecting fresh fecal pellets and plating homogenates on YPD-SA. All uninoculated mice remained uncolonized by C. albicans throughout the experiment.

Prior to clindamycin challenge, mice were divided into standard-size cages of 2 or 3 mice each. Fecal pellets were collected for sequencing on the day before clindamycin challenge (day −1, Fig. 1A). Each mouse was given a single intraperitoneal injection of clindamycin. Across the two trials, 6 uncolonized mice received the low dose (1.11 mg/kg body weight) of clindamycin, 7 received the intermediate dose (3.33 mg/kg), and 7 received the high dose (10 mg/kg); there were 8 C. albicans-colonized mice in each clindamycin treatment group. Fecal pellets were collected for sequencing 1, 3, and 5 days after clindamycin challenge. Mice were sacrificed on day 7 post-clindamycin challenge.

All experiments were conducted in compliance with the NIH Guide for the Care and Use of Laboratory Animals and Tufts University IACUC guidelines.

### Microbiota analysis.

DNA was extracted from fecal pellets using the QIAamp DNA stool minikit (Qiagen 51504, discontinued) with RNase/proteinase K digestion. Briefly, frozen fecal pellets were homogenized in lysis buffer, further disrupted through bead beating and incubated with an InhibitEX tablet. The supernatant was then digested with RNase (1 mg/ml) and proteinase K (20 mg/ml), and DNA was isolated using column purification. Some samples were prepared using the QIAamp PowerFecal Pro DNA kit (Qiagen 51804) according to the manufacturer’s protocol. Libraries were prepared from each DNA sample and sequenced as described previously (59). Briefly, PCR amplification of the V4 region of the 16S rRNA gene was performed with primers that included adapters for Illumina sequencing and 12-base barcodes; 250-bp paired-end sequencing was performed using an Illumina MiSeq. Base calling was performed using CASAVA 1.8, and the resulting fastq files were used as input for downstream analysis using QIIME2 (2018.8) (60). Briefly, the paired-end reads from the fastq files were joined, barcodes were extracted, and the reads were demultiplexed. Quality filtering and denoising using default parameters were performed using DADA2 (61). Operational taxonomic units (OTUs) were aligned using mafft (62) via q2-alignment and used to construct a phylogenetic tree using fasttree2 (63). Standard alpha diversity and beta diversity (using weighted UniFrac) metrics were calculated using q2-diversity plugin after rarefaction (subsampling without replacement) at the depth of 23,000 sequences per sample. Taxonomy was assigned using the q2-feature-classifier (64) against the Greengenes 13_8 99% OTU reference sequences (65). Longitudinal analysis was performed using q2-longitudinal (66). PERMANOVA was performed with QIIME2 with the default 999 permutations. All other statistical analyses of microbiota data were performed in Prism.

Microbiota taxonomic analysis output from QIIME2 was used to define the relative abundance of different genera within each mouse microbiota. qPCR using universal primers for bacterial 16S rRNA (primers ACTCCTACGGGAGGCAGCAGT and TATTACCGCGGCTGCTGGC [67]) was used to measure the total amount of bacterial DNA present in each sample and was normalized to the weight of fecal pellet used for DNA extraction. Relative abundance was multiplied by total bacterial DNA (arbitrary units) (see Fig. S1A in the supplemental material) to quantify absolute abundance at the genus level.

For measurement of fold change in response to clindamycin treatment, the absolute abundance of each genus after treatment (day 1) was divided by the absolute abundance before treatment (day −1). Individual mice with undetectable levels of a genus before treatment were omitted from this analysis.

### Network analysis.

Microbiota sequencing data from the pre-clindamycin, day −1, fecal pellets were used to perform network analysis. Absolute abundance data were used for correlation analysis in R to determine correlation between every genus and every other genus within each individual microbiota sample. All genera detected with a median absolute abundance greater than zero prior to clindamycin challenge were included. Network analysis was performed in R using igraph (1.2.5) (39) to generate the co-occurrence network and perform fast-greedy cluster analysis and qgraph (1.6.5) (40) to measure network topography, including density, modularity, and centrality.

### Statistical analysis.

Statistical analysis was largely performed in Prism. Experimental groups were compared using the Student’s *t* test or Welch’s *t* test (when variance was significantly different). When comparing more than two experimental groups, groups were compared using ANOVA followed by Sidak’s *post hoc* test adjusted for multiple comparisons. The DiffCorr (0.4.1) (44) package in R was used to compare correlation coefficients of the uncolonized and C. albicans-colonized networks using Fisher’s Z-test followed by the Benjamini-Hochberg correction for multiple comparisons. Throughout, a significance cutoff α value of 0.05 was used.

### Data availability.

Microbiota sequencing data generated by this project are available in the NCBI BioProject database under accession no. PRJNA690557.
