# Supplementary material for: Decreased Ecological Resistance of the Gut Microbiota in Response to Clindamycin Challenge in Mice Colonized with the Fungus Candida albicans
Source: mSphere. 2021 Jan 20;6(1):e00982-20. doi: 10.1128/mSphere.00982-20 (PMC7845615; doi:10.1128/mSphere.00982-20)
Supplement: TABLE S1 [file mSphere.00982-20-st001.docx]

# Table S1: Complete list of statistically significant differential correlations from DiffCorr

| Genus | Genus | R: Uncol | R:  *C. albicans* | P-value (difference) |
| --- | --- | --- | --- | --- |
| *Akkermansia* | *Dorea* | 0.85 | -0.76 | 1.55E-09 |
| *Akkermansia* | *Allobaculum* | 0.87 | -0.71 | 1.55E-09 |
| *Akkermansia* | Unclassified S24-7 | -0.50 | 0.92 | 5.99E-09 |
| *Akkermansia* | Erysipelotrichaceae *Clostridium* | 0.78 | -0.70 | 3.31E-07 |
| *Akkermansia* | *Bifidobacterium* | 0.80 | -0.63 | 9.62E-07 |
| Unclassified Ruminococcaceae2 | *Bacteroides* | 0.91 | -0.16 | 1.20E-05 |
| *Oscillospira* | Unclassified Lachnospiraceae2 | 1.00 | 0.82 | 2.62E-04 |
| Clostridiaceae *Clostridium* | *Bacteroides* | 0.87 | -0.12 | 3.71E-04 |
| *Akkermansia* | Unclassified Christensenellaceae | 0.58 | -0.64 | 4.39E-04 |
| *Akkermansia* | Unclassified Erysipelotrichaceae | 0.81 | -0.27 | 5.01E-04 |
| *Butyricicoccus* | *Bacteroides* | 0.82 | -0.22 | 5.49E-04 |
| *Akkermansia* | *Sutterella* | 0.66 | -0.53 | 5.49E-04 |
| Ruminococcaceae *Ruminococcus* | *Bacteroides* | 0.80 | -0.12 | 3.94E-03 |
| Unclassified Ruminococcaceae2 | Unclassified Clostridiales2 | 0.49 | 0.94 | 4.96E-03 |
| *Allobaculum* | *Bifidobacterium* | 0.94 | 0.53 | 6.30E-03 |
| Unclassified Clostridiales | *Bacteroides* | 0.66 | -0.35 | 8.33E-03 |
| *Akkermansia* | Lachnospiraceae *Ruminococcus* | 0.33 | -0.66 | 8.39E-03 |
| Unclassified Lachnospiraceae2 | Unclassified Christensenellaceae | 0.24 | 0.87 | 1.22E-02 |
| *Akkermansia* | Unclassified Lachnospiraceae | 0.19 | -0.72 | 1.22E-02 |
| Lachnospiraceae *Ruminococcus* | *Bacteroides* | 0.71 | -0.20 | 1.42E-02 |
| *Akkermansia* | Unclassified Ruminococcaceae2 | 0.47 | -0.50 | 1.79E-02 |
| *Butyricicoccus* | Unclassified Clostridiales2 | 0.49 | 0.92 | 1.95E-02 |
| Unclassified Ruminococcaceae | *Coprococcus* | -0.01 | 0.78 | 1.95E-02 |
| *Allobaculum* | Unclassified Erysipelotrichaceae | 0.82 | 0.11 | 1.95E-02 |
| Unclassified Mogibacteriaceae | Unclassified Christensenellaceae | 0.32 | 0.87 | 2.95E-02 |
| *Butyricicoccus* | *Coprococcus* | 0.93 | 0.57 | 2.95E-02 |
| Lachnospiraceae *Ruminococcus* | Unclassified Lachnospiraceae2 | 0.63 | 0.94 | 3.03E-02 |
| Unclassified Christensenellaceae | *Bacteroides* | 0.59 | -0.29 | 3.11E-02 |
| Unclassified Lachnospiraceae | *Bacteroides* | 0.68 | -0.15 | 3.11E-02 |
| Unclassified Lachnospiraceae | Unclassified Clostridiales | 0.97 | 0.80 | 3.11E-02 |
| Lachnospiraceae *Ruminococcus* | Unclassified Christensenellaceae | 0.38 | 0.88 | 3.11E-02 |
| Ruminococcaceae *Ruminococcus* | Unclassified Lachnospiraceae | 0.95 | 0.69 | 3.11E-02 |
| *Allobaculum* | Unclassified Lachnospiraceae2 | -0.05 | 0.73 | 3.11E-02 |
| *Akkermansia* | Unclassified Mogibacteriaceae | 0.12 | -0.69 | 3.11E-02 |
| Unclassified Mogibacteriaceae | Lachnospiraceae *Ruminococcus* | 0.65 | 0.94 | 3.40E-02 |
| *Akkermansia* | *Coprococcus* | 0.44 | -0.42 | 4.25E-02 |
| Unclassified Ruminococcaceae2 | Unclassified Lachnospiraceae2 | 0.59 | 0.92 | 4.69E-02 |
| *Dorea* | *Bacteroides* | 0.73 | 0.02 | 4.73E-02 |
| *Allobaculum* | Unclassified Ruminococcaceae | -0.08 | 0.68 | 4.73E-02 |
